# Effects of Computerized Updating and Inhibition Training in Older Adults: The ACTOP Three-Arm Randomized Double-Blind Controlled Trial

**DOI:** 10.3389/fneur.2020.606873

**Published:** 2020-12-03

**Authors:** Arnaud Boujut, Lynn Valeyry Verty, Samantha Maltezos, Maxime Lussier, Samira Mellah, Louis Bherer, Sylvie Belleville

**Affiliations:** ^1^Research Center, Institut Universitaire de Gériatrie de Montréal, Montreal, QC, Canada; ^2^Psychology Department, Université de Montréal, Montreal, QC, Canada; ^3^Department of Medicine, Université de Montréal, Montreal, QC, Canada; ^4^Research Center, Institut de Cardiologie de Montréal, Montreal, QC, Canada; ^5^Department of Neuroscience, Université de Montréal, Montreal, QC, Canada

**Keywords:** cognitive training, attentional control, working memory, transfer, aging

## Abstract

**Background:** Working memory (WM) capacity declines with advancing age, which impacts the ability to carry out complex cognitive activities in everyday life. Updating and inhibition processes have been identified as some of the most critical attentional control processes of WM and are linked to age-related WM decline. The general aim of the Attentional Control Training in Older People (ACTOP) study was to perform a side-by-side comparison of updating and inhibition training to examine their respective efficacy and transfer in cognitively healthy older adults.

**Method:** The study was a three-arm, double-blind, randomized controlled trial registered with the US National Institutes of Health clinical trials registry. Ninety older adults were randomly assigned to 12 half-hour sessions of updating (N-back type exercises), inhibition (Stroop-like exercises) computerized training or active control (general knowledge quiz game). A group of thirty younger adults completed all proximal and WM transfer tasks without training to assess age-related deficits prior to training and whether training reduces these deficits.

**Results:** Piecewise mixed models show quick improvement of performance during training for both updating and inhibition training. During updating training, the progression was more pronounced for the most difficult (3-back) than for the least (1-back) difficult level until the ninth session. Updating and inhibition training groups improved performance on all proximal and WM transfer measures but these improvements did not differ from the active control group. Younger adults outperformed older ones on all transfer tasks prior to training. However, this was no longer the case following training for two transfer tasks regardless of the training group.

**Conclusion:** The overall results from this study suggest that attentional control training is effective in improving updating and inhibition performance on training tasks. The optimal dose to achieve efficacy is ~9 half-hour sessions and the dose effect was related to difficulty level for updating training. Despite an overall improvement of older adults on all transfer tasks, neither updating nor inhibition training provided additional improvements in comparison with the active control condition. This suggests that the efficacy of process-based training does not directly affect transfer tasks.

**Clinical Trial Registration:** www.ClinicalTrials.gov, identifier: NCT03532113

## Introduction

Age-related cognitive decline is associated with a higher risk of pathological aging and loss of autonomy. Cognitive training programs are non-pharmacological interventions that have the potential to increase cognition in older adults and reduce age-related cognitive decline (1). Working memory (WM) is a target of major interest for cognitive training because it is a critical component of high-level cognitive processes that are needed to adapt to complex situations in daily life. WM is also of particular interest in the context of aging because WM performance declines with advancing age (2), which reduces autonomy (3). Importantly, WM is not a monolithic process and involves various fine-tuned attentional control components [see Engle (4) for a review], which can be the target of different types of training programs. In particular, updating and inhibition have been identified as some of the most critical attentional control processes of WM (5), and are linked to age-related WM decline (6). According to Sylvain-Roy's et al. (6) results, updating and inhibition are the control processes that best predict WM preformance in aging.

Updating and inhibition are relatively independent processes that rely on different brain systems [e.g., (7, 8)]. There are some data showing that both updating and inhibition training can improve performance on trained tasks in older adults [see Nguyen et al. (9) for a meta-analysis]. However, their relative efficacy may differ and no study has yet conducted a side-by-side comparison of their respective effect. Furthermore, to examine training efficacy, it is necessary to determine whether the training dose is sufficient to elicit an improvement in the target process and whether the training effect is more pronounced at the highest level of task difficulty when the target process is most needed. The relationship between the level of training difficulty and training dose is generally overlooked in cognitive intervention studies.

One other critical question relates to the impact of updating and inhibition training on transfer tasks. Some studies have found proximal transfer, where improvements following updating training were transferred to an untrained updating task in older adults [e.g., (10–13)] and training-related improvements in inhibition, which were transferred to an untrained inhibition task (8, 14). However, proximal transfer is not systematically observed in older adults (7, 14, 15). Moreover, it is unclear whether training effects lead to distal transfer to complex WM tasks that may rely on trained processes. As complex WM tasks are hypothesized to rely on updating and inhibition, transfer is expected. No study has reported transfer to complex WM tasks for inhibition and only one study found WM transfer of updating training in older adults (16), while other studies have reported no transfer (7, 15, 17). Finally, in cases where a training effect is found, it is unknown whether the increase in performance is large enough to remove the performance difference typically observed between older and younger adults.

The general aim of the Attentional Control Training in Older People (ACTOP) study was to perform a side-by-side comparison of updating and inhibition training in a randomized double-blind controlled trial to examine their respective efficacy and their transfer to proximal and complex WM tasks in cognitively healthy older adults. The first objective was to determine the relationship between cumulative training dose and improvement on the trained exercises at different levels of task demand. The second objective was to examine whether such an improvement transferred to proximal untrained tasks and to complex WM tasks over and beyond what was observed following an active control training condition. As the ultimate goal is to observe a transfer effect in everyday life, transfer to complex WM was not only assessed with typical WM tasks but also using a virtual reality WM task mimicking real-life situations (18, 19). The third objective was to assess whether the training programs reduced or eliminated the age difference between older and younger adults. This was done by evaluating the age difference by comparing older adults to a group of untrained younger adults prior and after training. We expected, (1) a comparable non-linear dose-response function in the updating and inhibition trained tasks, with a larger slope during the initial phase of the training for the most difficult exercises, and (2) a larger effect on proximal and WM transfer tasks for the updating and inhibition than active control training condition. According to Sylvain-Roy's et al. results, (6), (3) updating training should lead to a more pronounced transfer to the WM reading span task, and inhibition training should lead to a more pronounced transfer to the WM alpha span task. (4) We also expected an age effect at baseline, which should be no longer be found after updating or inhibition training in older adults.

## Methods

### Design

**Figure 1:**
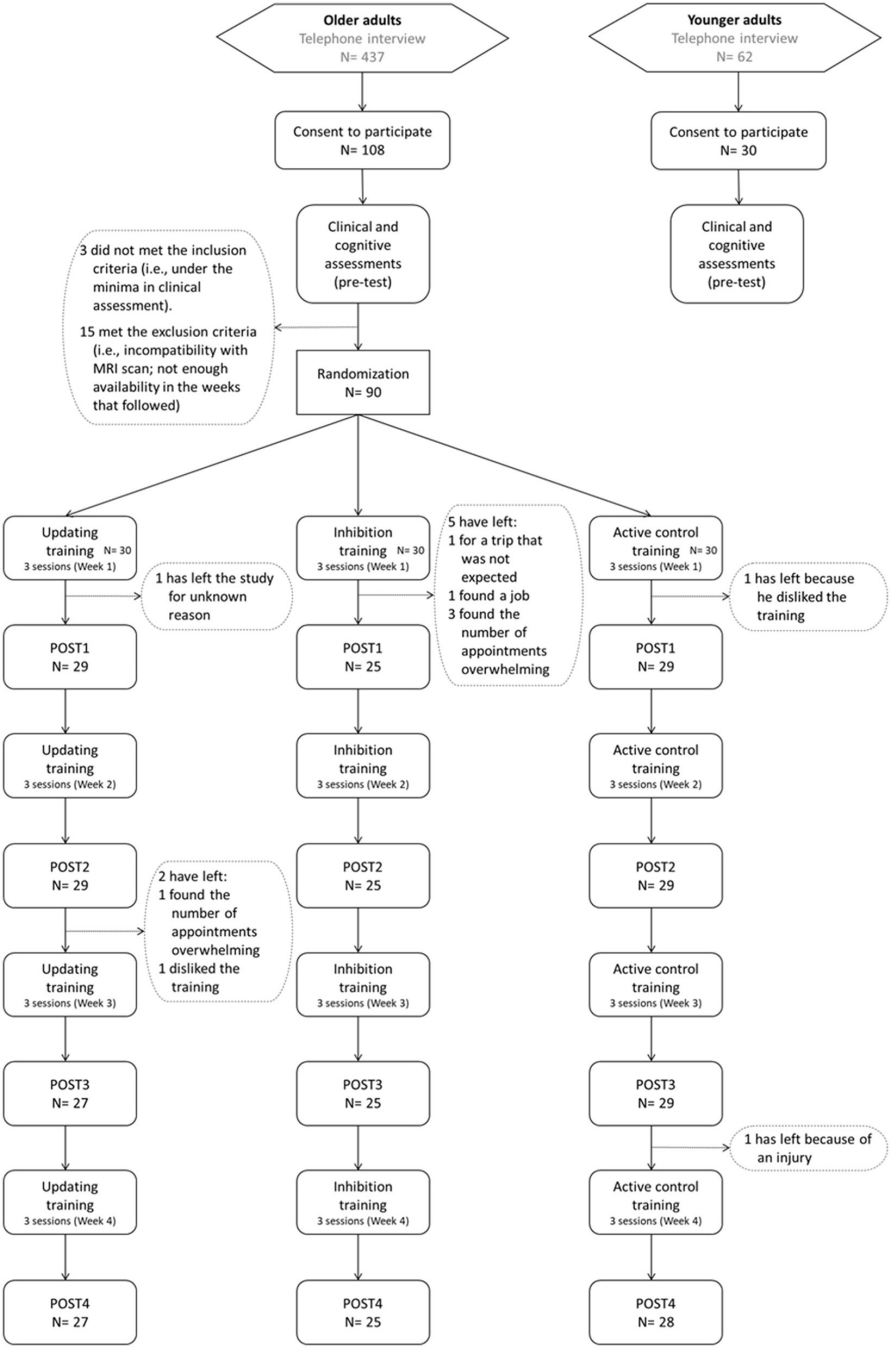
Flow diagram indicating participant progress throughout the trial.

The ACTOP cognitive training study was a three-arm randomized double-blind controlled trial completed in French at the Center de recherche de l'Institut universitaire de gériatrie de Montréal (CRIUGM) and registered as a clinical trial with the US National Institutes of Health clinical trial registry (ClinicalTrials.gov Identifier: NCT03532113). Participants were randomized to updating, inhibition or general knowledge (active control) interventions (see Figure 1). A project coordinator, who was not involved with the enrolment process, cognitive assessments or interventions, performed randomization using a computer-based random number generator (i.e., one participant at a time as they entered the study). Training was computerized and completed individually under the supervision of a trainer at the research center in groups of six to 10 participants. Although the small groups were not organized by type of intervention but were mixed to accommodate participants' schedules, participants were unaware of the experimental and control conditions, and were told that different capacities were trained. The distribution of participants among the interventions was known by the training supervisors while the assessors were blind. There was one PRE-intervention (PRE) and four post-baseline assessments performed throughout the 4 weeks of training sessions [after 3 sessions (POST1), 6 sessions (POST2), 9 sessions (POST3), and 12 sessions (POST4) of training]. The research conformed with the ethical rules for human experimentation stated in the Declaration of Helsinki and was approved by the Comité d'éthique de la recherche Vieillissement et Neuroimagerie, Center intégré universitaire de santé et de services sociaux (CIUSSS) du Center-Sud-de-l'Île-de-Montréal (application #CERVN17-18-02, approval 8 May 2017).

### Participants

Our goal was to recruit a total of 90 participants. Considering an attrition rate of about 10% based on our prior experience [e.g., (20)], ~27 participants should be allocated per condition. Assuming a significance level of α = 0.05, a power of 0.80 and a correlation of *r* = 0.50 between three repeated measures, the G*Power 3 software for mixed designs estimates that the sample size will provide sufficient power to detect a small to medium effect (*f* = 0.15). This corresponds well to the effect sizes reported for similar training programs [see Lampit et al. (21) for a review]. Thus, the study was designed to be powered to detect the expected effect size. The 90 older adults (age 60–85) and 30 younger adults (age 20–35) were recruited in the Montreal area through local newspapers, associations, community centers and CRIUGM's participant registry (*Banque de participants du CRIUGM*). Neuropsychological and baseline tests were performed before randomization (see Table 1). Participants were enrolled in the study if they showed no memory impairment by scoring above the cut-offs on the delayed recall portion of the Logical Memory test of the Wechsler Memory Scale for older adults. For additional details on baseline tests and inclusion and exclusion criteria, see Boujut et al. (22).

**Table 1:** Variables and tests used in the study.

**Variables and test**	**Type of tests**			**Timeline of measurement**				
	**Clinical assessment**	**Proximal transfer**	**WM transfer**	**Pre-test**	**POST1**	**POST2**	**POST3**	**POST4**
Age				x				
Sex				x				
Montreal cognitive assessment (MoCA)	x			x				
Logical memory test	x			x				
Geriatric depression scale (GDS)	x			x				
Ischemic index	x			x				
Cognitive reserve proxy-questionnaire				x				
**Updating composite measure**		x						
Keep track z-score		x		x		x		x
Running span z-score		x		x		x		x
**Inhibition composite measure**		x						
Stroop victoria z-score		x		x		x		x
Anti-saccade z-score		x		x		x		x
**Complex working memory**								
Alpha span			x	x	x	x	x	x
Reading span			x	x	x	x	x	x
Dual virtual reality task measure								
Verbal memory z-score			x	x	x	x	x	X
Visual detection z-score			x	x	x	x	x	X

### Interventions

**Figure 2:**
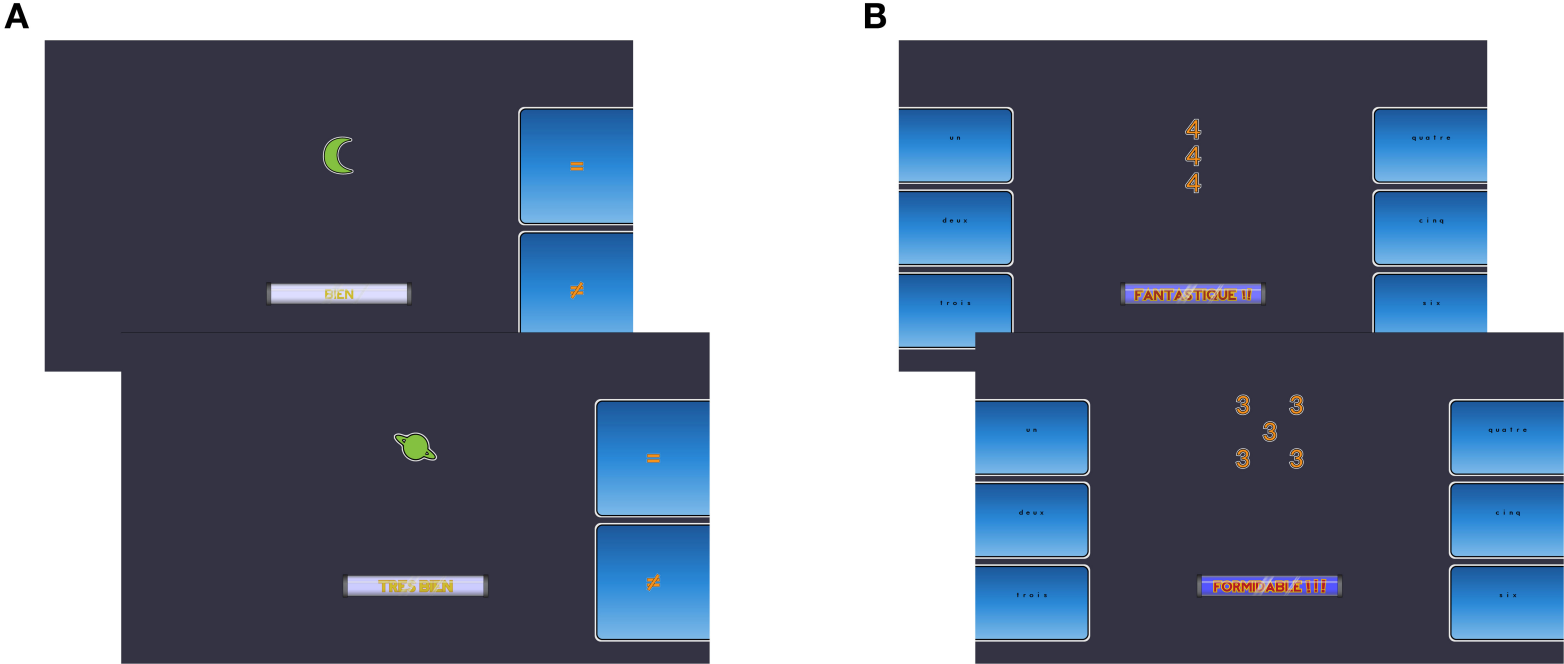
Illustration of the attentional control training. **(A)** Example of an N-back exercise used for updating training. In this 1-back trial, the planet symbol does not match the previously displayed moon symbol. **(B)** Two examples of a Stroop-like exercise used for inhibition training. In these incongruent trials, the correct response is “three” for the example shown above and “five” for the example below.

The updating and inhibition computerized training programs were provided by the Neuropeak web platform [Lussier et al. Normative data for a tablet-based executive function assessment battery in healthy older adults, forthcoming; see also (23–25)], and was completed on a Samsung Galaxy Tab 2 (Android version 4.2.2). Figure 2 illustrates the layout for the updating (Figure 2A) and inhibition training (Figure 2B). The general knowledge quiz (active control) was presented with E-Prime 3.0 software on a laptop (Lenovo). The three interventions were provided three times per week for 4 weeks (i.e., 12 training sessions) under the supervision of a trainer helping the participants manage technical issues and encouraging completion of all exercises.

#### Updating Training

Each updating training session comprised two N-back type exercises consisting of digits (1 to 9) or symbols (moon, planet, star, dog, bird, snake) (see Figure 2A for an illustration of the N-back type exercise for updating training using symbols). Each exercise included three difficulty levels (1-back, 2-back, 3-back) delivered over eight blocks in the following order: 1-back (two blocks of 11 trials), 2-back (three blocks of 12 trials), and 3-back (three blocks of 13 trials). Participants were asked to indicate whether each item matched the one previously presented in the *n* position (e.g., 6–5–6–8–5 wherein the second “5” is the only match in a 3-back block). Each block included 40% “match” responses. The access to the 3-back level was conditional on achieving a minimum of 75% accuracy at the 2-back level. If this minimum was not reached, participants finished the session with a 1-back block. “Match” and “Mismatch” buttons were displayed on the right side of the screen and the right thumb was used to respond. Participants were instructed to respond as accurately as possible within a 3-second time limit.

#### Inhibition Training

Each inhibition training session comprised two Stroop-like exercises using compound stimuli made of digits (1 to 9) or letters (D, F, H, L, S, T) (see Figure 2B for an illustration of the Stroop-like exercise for inhibition training using digits). In the first case, participants were instructed to count the number of items in each trial. In the second case, participants were instructed to identify the larger letter, which was formed by smaller letters or symbols. Each exercise included three difficulty levels (congruent, neutral, incongruent) delivered over seven blocks in the following order: (1) 20 congruent stimuli (e.g., five copies of the digit “5” or a large “H” formed from smaller Hs), (2) 60 neutral stimuli (e.g., five copies of the symbol “*” or a large “H” formed from smaller “*”), (3) 60 incongruent stimuli (e.g., five copies of the digit “3” or a large “L” formed from smaller Hs), (4) 20 congruent stimuli, (5) 60 incongruent, (6) 20 congruent, and (7) 60 incongruent stimuli. Incongruent stimuli were expected to require inhibition processes. However, congruent and neutral stimuli were also expected to be important to reduce stimulus-response dependency. The participants responded by using their thumbs to press the response keys located on each side of the touchscreen.

#### Active Control Training

Each general knowledge quiz training session comprised two blocks of 40 new multiple-choice questions, consisting of four options. The questions were adapted from https://www.openquizzdb.org/index.php or created by our research team. The quiz covered ~18 different topics, including food, science, geography, video games, history, sports, music, inventions, animals, movie and television series, art and literature, Canada, physics and space, monuments in the world, key historical dates, people and languages, herbs and spices, and fruit trees. Participants responded by pressing the corresponding answer on a keypad (the answer was numbered 1, 2, 3, and 4).

### Measures

#### Efficacy Based on Trained Measures

For updating and inhibition training, the dependent measure was the Inverse Efficiency Score (IES), which corresponds to mean reaction time divided by mean proportion of accuracy. Only reaction times under 4,000 ms were used as valid responses. In updating training, the IES was calculated separately for each difficulty level for each of the 12 training sessions. It was based on 10 trials per block regardless of match/mismatch distinction. As the first trials (i.e., the first 1–2–3 trials from the 1-, 2-, and 3-back blocks, respectively), do not have match responses, they were not used for the IES caculation. In inhibition training, IES was calculated separately for congrurent and incongruent stimuli in each session. IES from neutral stimuli were not analyzed because there were only two blocks per session (one block per exercice). IES was based on 20 trials per block. Even though the number of trials was greater in the incongruent (60 trials) than congruent blocks (20 trials), IES was calculated for trials with equivalent positions in their respective blocks (i.e., trials from 1 to 20). In order to examine the pattern of improvement during training, the analyses will split the growth curves in successive segments.

#### Proximal Transfer

To evaluate the proximal transfer to untrained updating and inhibition tasks, a composite updating score was calculated by averaging the z-scores [z = (x–x_*PRE*_)/s_*PRE*_] from the keep track task (26) and the running span task on PRE-training, POST2 and POST4. A composite inhibition score was calculated by averaging the z-scores from the Victoria Stroop task (27) and the anti-saccade task (28) on the same time points. The measurements were limited to three time points due to the number of associated tasks.

In the keep track task, words from four different categories (e.g., fruits, clothes, music, colors) were displayed one by one on a computer screen. Each time participants encountered a new word from the same category, they were asked to keep it in mind so that they could recall the last word in each of the four categories when the list ended. The dependent variable was the proportion of words correctly recalled. In the running span task, lists of letters were displayed one by one on a computer screen. Participants were asked to report the *n* last letters in their correct order (*n* = span size minus 1), but were not informed of the list's length in advance. The dependent variable was the span-adjusted proportion of letters correctly recalled.

In the Victoria Stroop task, participants were asked to name colors of dots printed in color, non-color words printed in color, and finally, the colors of the printed words, which were a different color than the word. The dependent variable was the reading time for the incongruent colored words divided by the reading time for the dots printed in color. In the anti-saccade task, participants were asked to indicate the pointing direction of an arrow (up or down) presented in the right or left portion of a computer screen. Prior to the arrow presentation, a flashed cue appeared on the opposite side of the screen as a distraction. Participants provided their response by pressing a key. The dependent variable was the proportion of correctly identified target arrow directions, despite the distracting cue.

#### Transfer to Complex WM Tasks

Based on the Sylvain-Roy's et al. (6) study, transfer to complex WM tasks was measured using the alpha-span task (29) and reading span task (30). In addition, an immersive dual virtual reality task (19) was used to reproduce complex real-life situations. Measurements were taken on PRE-training, POST1, POST2, POST3, and POST4.

In the alpha-span task, participants were asked to orally recall series of words in alphabetical order rather than in the order of presentation. The size of the series corresponded to *n* minus 1 (*n* = span size). The dependent variable was the span-adjusted proportion of words recalled in the correct order.

In the reading span task, participants made yes/no semantic plausibility judgments on a series of two to five sentences. Following each series, participants were asked to orally recall the last word of each sentence. The dependent variables are the proportion of correct words recalled.

In the dual virtual reality task, participants wore an HMD nVisor ST50 headset with stereoscopic vision (1,280 × 1,024 full color with 50° diagonal field-of-view), which allowed them to be a passenger in a virtual car ride on a highway. Participants were asked to guide the driver to a fictitious city (“Chauminont” or “Montformeil”) by pressing a mouse button each time they saw a road sign indicating the city in question. While they were doing the guiding task, they were asked to memorize and recall two series of twelve words that were presented orally by the driver. The dependant variable was a score combining the z-scores from the number of correct detections for the road sign task and the number of correct recalls on the memory task.

### Data Analysis

We first analyzed demographic information (age, sex, education) and baseline characterization from the cognitive reserve proxy questionnaire (CRQ) (31), ischemic index (32) and depression questionnaires [short version of Geriatric depression scale (GDS) for older adults (33) and Beck Depression Inventory II (BDI-II) for young adults]. Cognition was measured with the Montreal Cognitive Assessment (MoCA) (34) and a French version of the logical memory subtest (CIMAQ) (35) adapted from the Wechsler Memory Scale (36) using separated one-way ANOVAs with Group (three levels) as a between-subject factor.

Then, a modified intention-to-treat (mITT) approach was used, where participants were retained for analysis if they completed at least one post-baseline assessment. Current guidelines [e.g., Schulz et al. (37) for the CONSORT] emphasize the importance of relying on intention-to-treat (ITT) principles. Accordingly, and as was done here, studies should fully report deviations from treatment allocation, and missing data, recognizing that attrition is observed in most trials. Furthermore, the guidelines suggest relying on analytic models that are resistant to missing values and can make use of the full data set. This was done here by using mixed linear models to analyze the efficacy and transfer data. However, relying on full ITT analyses has been criticized because it reduces the ability to test the true efficacy of a treatment if non-treated individuals (i.e., with data only at baseline) are included as treated. In such cases, it is justified to use a subset of the ITT population (mITT) as was done here (38). Our retention of participants with at least one data point also reduces the detrimental impact of mixed linear models using degrees of freedom based on the number of participants tested only. Analyses were performed with R software (Version 3.6.1) using the *nlme* R package for linear mixed-effects models (LMMs). Marginal *R*^*2*^ was calculated using the *MuMIn* package to describe the proportion of variance explained by the fixed factor(s).

Efficacy and effect of the training dose was examined on the growth curves (i.e., IES) using two steps. Step 1 consisted of a building process of unconditional growth models (i.e., linear growth model, quadratic growth model, and piecewise growth model) to select the best fit model over time using maximum likelihood estimation and an autoregressive covariance structure (corAR1). The time scores for the slope growth factor were coded as a continuous variable from 0 (i.e., the 1st training session) to 11 (i.e., the 12th session). For the piecewise growth model, time scores were segmented into four segments of three training sessions. Such time segmentation may not be the most parsimonious segmentation for a piecewise growth model. However, this choice was justified by the interest in specifying the role of each training segment in the performance growth. Preliminary analyses showed that the piecewise growth model fit the data better than other models for the updating and inhibition trained tasks (see below). Model fit was based on the chi-square test but the Akaike Information Criterion (AIC) and Bayesian Information Criterion (BIC) were also reported in Appendix 1 based on Meteyard and Davies' (39) template. Step 2 consisted of a series of conditional growth models examining the effect of time for a set training period as a function of difficulty level. Due to the presence of extreme IES values, trials that were more than three standard deviations (SDs) away from a session's mean IES for the same difficulty level were discarded. Eight out of 879 values were discarded in the updating condition and 19 out of 1,199 in the inhibition condition.

The effect on the transfer tasks were also examined following a model building process. However, given the small number of measurement points over time (from three to five), the objective was not to determine the shape of the performance growth but whether participants had a linear slope that varied by intervention group. The time scores were coded as continuous variables starting from 0 (PRE) to 4 (POST4).

Finally, age effects were assessed on the transfer tasks with separate analyses of variance (ANOVAs) for PRE and POST4 using group as a between-subject factor. Tukey *post hoc* tests were performed using the *emmeans* package to compare the older groups to the younger group within each condition, but also to compare training conditions within each age group.

## Results

### Baseline Characteristics and Age Group Comparison

ANOVAs showed that the training groups did not differ at baseline in terms of their clinical, cognitive and demographic characteristics (see Table 2). The group of younger adults was equivalent to the three groups of older adults for education and CRQ scores. The proportion of females was 0.76, 0.80, 0.76, and 0.70 in the updating, inhibition, active control and young adult groups, respectively, and there was no significant difference between groups [χ2(3) = 0.76, *p* = 0.86]. As expected, younger participants outperformed older adults on cognitive measures with the exception of the immediate recall from the logical memory test [*F*(3, 109) = 1.62, *p* > 0.1], the control condition from the delayed logical memory subtest (*post hoc* comparison: *p* > 0.1), the updating and control conditions from the MoCA (*p* > 0.1 and *p* > 0.05, respectively), and the updating condition from the visual detection sub-task from the dual virtual reality task (*p* > 0.1).

**Table 2:** Participant's clinical characteristics and group comparisons at baseline (PRE).

	**Updating intervention**	**Inhibition intervention**	**Active control intervention**		**Young adults**	
**Characteristic**	***n* = 29**	***n* = 25**	***n* = 29**	***p*-values**	***n* = 30**	***p*-values**
**Demographic**						
Sex, *n*						
Male	7	5	7		9	
Female	22	20	22		21	
Age, mean ± SD	68.1 ± 5.6	70.8 ± 5.1	71.3 ± 5.9	0.07	27.1 ± 4.7	0.00^***^
Education, years, mean ± SD	14.4 ± 2.3	14.8 ± 2.6	14.6 ± 2.6	0.82	15.0 ± 2.4	0.80
**Clinical assessment**
Montreal cognitive assessment (MoCA) (range 0–30)	28.1 ± 1.6	27.4 ± 1.6	27.9 ± 1.4	0.17	28.8 ± 1.5	0.01^**^
Logical memory test (range 0–25)
Immediate	15.3 ± 4.0	15.8 ± 3.3	16.2 ± 4.3	0.76	17.6 ± 3.7	0.17
Delayed	14.0 ± 3.9	14.1 ± 3.1	15.3 ± 4.3	0.38	17.3 ± 3.3	0.01^**^
Cognitive reserve questionnaire	18.6 ± 5.3	18.2 ± 4.4	18.5 ± 3.3	0.94	20.1 ± 3.0	0.27
Hachinski (range 0–18)	0.3 ± 0.6	0.5 ± 1.0	0.7 ± 0.8	0.21		
Geriatric depression scale (GDS) (range 0–15)	1.7 ± 2.9	2.1 ± 2.2	3.0 ± 2.3	0.17		
BDI (range 0–63)					5.2 ± 2.6	
**Inhibition composite measure**	−0.3 ± 0.7	−0.3 ± 0.8	−0.3 ± 0.7	0.98	0.8 ± 0.4	0.00^***^
Victoria stroop interference index (3rd plate/1st plate)	2.2 ± 0.4	2.1 ± 0.6	2.1 ± 0.5	0.63	1.6 ± 0.3	0.00^***^
Anti-saccade (range 0–90)	53.5 ± 20.9	51.0 ± 21.3	49.4 ± 20.0	0.76	78.1 ± 12.8	0.00^***^
**Updating composite measure**	−0.2 ± 0.7	−0.2 ± 0.5	−0.3 ± 0.6	0.83	0.7 ± 0.8	0.00^***^
Keep track (range 0–24)	13.7 ± 3.0	13.8 ± 2.3	13.8 ± 2.7	0.99	16.2 ± 2.7	0.00^**^
Running span (adjusted accuracy rate)	0.6 ± 0.1	0.7 ± 0.1	0.6 ± 0.2	0.58	0.8 ± 0.2	0.00^***^
**Complex working memory**						
Alpha span (adjusted accuracy rate)	0.7 ± 0.1	0.7 ± 0.1	0.7 ± 0.1	0.75	0.9 ± 0.1	0.00^***^
Reading span (range 0–56)	34.5 ± 9.8	33.8 ± 6.8	31.85 ± 7.5	0.35	42.0 ± 7.9	0.00^***^
Dual virtual reality task composite measure	−0.1 ± 0.6	−0.3 ± 0.8	−0.4 ± 0.7	0.21	0.8 ± 0.6	0.00^***^
Divided attention verbal memory (range 0–12)	3.8 ± 1.1	3.5 ± 1.1	3.7 ± 1.3	0.67	5.3 ± 1.4	0.00^***^
Divided attention visual detection (range 0–20)	16.5 ± 4.4	14.7 ± 6.3	13.4 ± 6.4	0.14	19.0 ± 1.6	0.00^***^

*P-values for analysis of variance group effect. SD, standard deviation*.

** p < 0.01;

*** *p < 0.001*.

### Efficacy Based on Trained Measures

**Figure 3:**
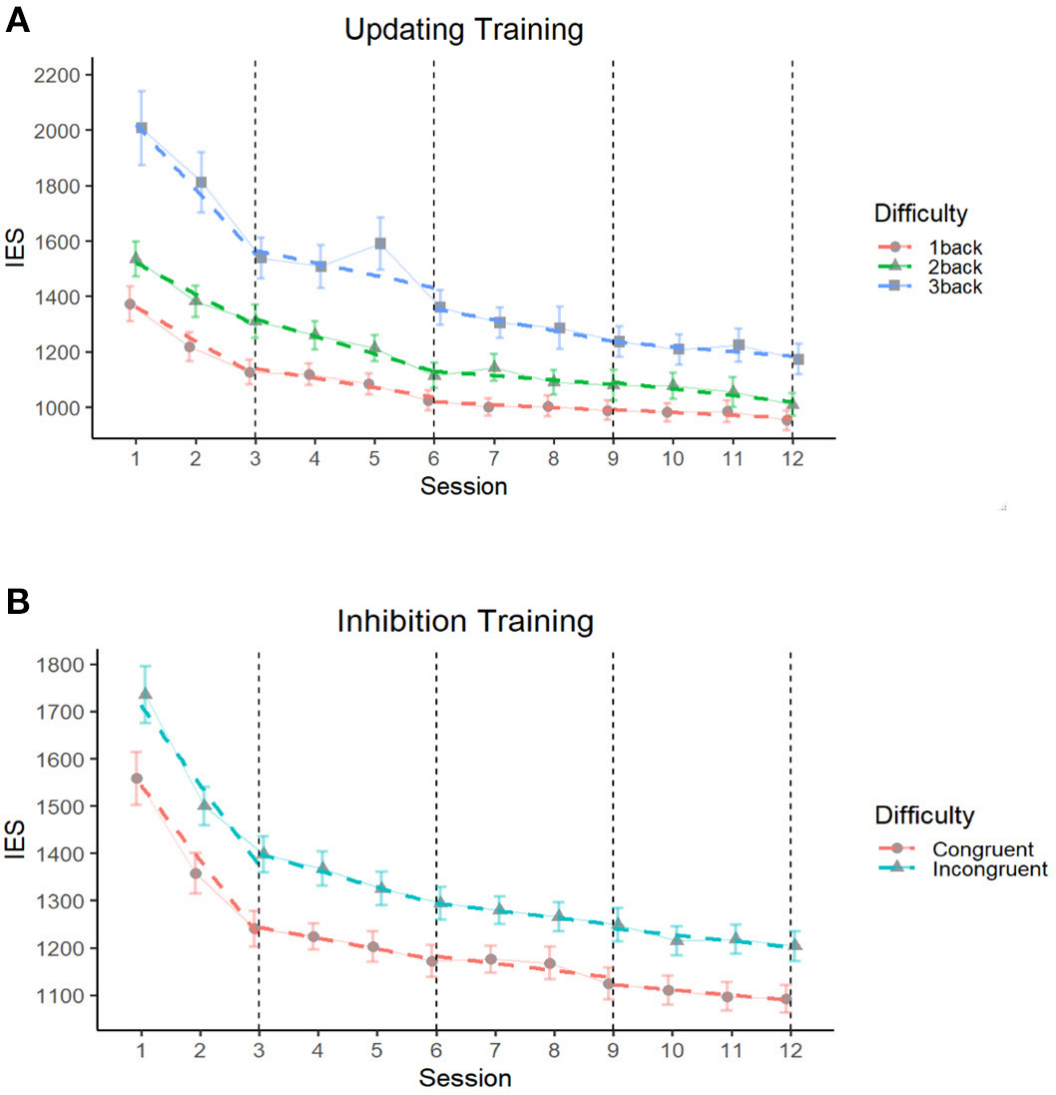
Comparison of performances (IES) by difficulty level throughout updating **(A)** and inhibition **(B)** training programs segmented by groups of three sessions. The Inverse Efficiency Score (IES) corresponds to the mean reaction time from each training session divided by the proportion of accuracy. Solid lines correspond to the average performance and colored dashed lines correspond to the regression slopes segmented into four segments of three training sessions representing the four time segments of training. The dashed black vertical line depicts the end of each training segment, where the slopes are differentiated. The error bars depict the SEM.

The building process of unconditional growth models (i.e., linear, quadratic, and piecewise) revealed that the piecewise growth model best fit the data for both the updating [χ^2^(11) = 20.03, *p* < 0.05] and inhibition [χ^2^(11) = 104.14, *p* < 0.001] training conditions (see Appendix 1). Four 3-session segments were delineated (see Figure 3). Conditional growth models showed that the addition of the Difficulty fixed effect and interaction term between Time_segments and Difficulty both improved the fit of the model for the updating [χ^2^(2) = 397.93, *p* < 0.001 and χ^2^(8) = 78.11, *p* < 0.001, respectively], and inhibition training [χ^2^(1) = 156.87, *p* < 0.001 and χ^2^(4) = 11.77, *p* < 0.05, respectively].

For the updating training, the intercept was 1,360 IES [*b* = 1,360.4 (77.06), *p* < 0.001) and the average growth for the first time segment was−114 (*b* = −113.89 (29.14), *p* < 0.001; see Table 3]. The average growth was no longer significant for time segment 2 [*b* = −29.54 (16.07), *p* = 0.07], time segment 3 [*b* = −12.64 (14.81), *p* = 0.39] and time segment 4 [*b* = −11.44 (16.98), *p* = 0.50]. Overall, as expected, the 3-back blocks were the least successful [*b* = 656.84 (45.23), *p* < 0.001], followed by the 2-back blocks [*b* = 162.18 (46.76), *p* < 0.001]. The conditional piecewise growth model showed that the 3-back blocks had the steepest slope of improvement in the first [*b* = −100.48 (32.69), *p* < 0.01], and third time segments [*b* = −43.40 (21.22), *p* < 0.05] (*p* > 0.05 for time segments 2 and 4; see Figure 3A).

**Table 3:** Time segmented conditional piecewise growth model on IES in updating training.

	**Est/Beta**	**SE**	**95% CI**	***t***	***p***
**FIXED EFFECTS**					
Intercept	1,360.4	77.06	1,210.43–1,510.32	17.65	0.0000^***^
Time_Segment1	−113.89	29.14	−170.58–−57.19	−3.91	0.0001^***^
Time_Segment2	−29.54	16.07	−60.81–1.73	−1.84	0.0664
Time_Segment3	−12.64	14.81	−41.47–16.18	−0.85	0.3936
Time_Segment4	−11.44	16.98	−44.49–21.60	−0.67	0.5006
2-back_blocks	162.18	46.76	71.19–253.16	3.47	0.0006^***^
3-back_blocks	656.84	45.23	568.82–744.86	14.52	0.0000^***^
Time_Segment1 X 2-back_blocks	2.57	33.85	−63.29–68.43	0.08	0.9396
Time_Segment1 X 3-back_blocks	−100.48	32.69	−164.10–−36.87	−3.07	0.0022^**^
Time_Segment2 X 2-back_blocks	−21.9	21.29	−63.33–19.54	−1.03	0.3041
Time_Segment2 X 3-back_blocks	−28.31	20.89	−68.96–12.34	−1.36	0.1757
Time_Segment3 X 2-back_blocks	0.55	21.64	−41.56–42.66	0.03	0.9798
Time_Segment3 X 3-back_blocks	−43.40	21.22	−84.69–−2.11	−2.05	0.0411^*^
Time_Segment4 X 2-back_blocks	−15.86	24.86	−64.24–32.52	−0.64	0.5238
Time_Segment4 X 3-back_blocks	−7.12	24.19	−54.20–39.96	0.29	0.7685
			**Variance**	**S.D**.	**Correlation**
**RANDOM EFFECTS**					
Participant			145,815.51	381.86	
Time_Segment1			9,306.06	96.47	−0.76
Time_Segment2			1,394.46	37.34	−0.87
Time_Segment3			50.86	7.13	−0.74
Time_Segment4			122.9	11.09	−0.9
				**Marginal**	**Conditional**
**MODEL FIT** ***R**^**2**^*					
				0.38	0.77

Model equation: IES ~ (Time_Segment1 + Time_Segment2 + Time_Segment3 + Time_Segment4) ^*^ Difficulty_of_blocks, random = ~ Time_Segment1 + Time_Segment2 + Time_Segment3 + Time_Segment4 | Participants, corAR1(0, form = ~ 1 | Participants).

* p < 0.05;

** p < 0.01;

*** *p < 0.001*.

For inhibition training, the intercept was 1,545 IES [*b* = 1,545.28 (52.36), *p* < 0.001; see Table 4]. The average growth in the first time segment was −157 [*b* = −156.84 (15.01), *p* < 0.001]. The average growth was also significant on the second [*b* = −15.70 (6.67), *p* < 0.05], third [*b* = −17.55 (5.40), *p* < 0.01] and fourth time segments [*b* = −14.76 (5.75), *p* < 0.05], albeit on smaller slopes. Overall, as expected, participants were the least successful at the highest difficulty level (incongruent blocks) [*b* = 170.37 (17.37), *p* < 0.001]. However, slope did not differ as a function of condition in any of the time segments (all the *p* > 0.1; see Figure 3B).

**Table 4:** Time segmented conditional piecewise growth model on IES in inhibition training.

	**Est/Beta**	**SE**	**95% CI**	***t***	***p***
**FIXED EFFECTS**					
Intercept	1, 545.28	52.36	1,443.30–1,647.26	29.51	0.0000^***^
Time_Segment1	−156.84	16.19	−188.37–−125.31	−9.69	0.0000^***^
Time_Segment2	−15.70	6.67	−28.70–−2.70	−2.35	0.0190^*^
Time_Segment3	−17.55	5.40	−28.08–−7.03	−3.25	0.0012^**^
Time_Segment4	−14.76	5.75	−25.97–−3.55	−2.57	0.0106^*^
Incongruent_blocks	170.37	17.37	136.53–204.20	9.81	0.0000^***^
Time_Segment1 X Incongruent_blocks	−9.86	12.10	−33.43–13.72	−0.81	0.4158
Time_Segment2 X Incongruent_blocks	−12.70	7.78	−27.85–2.46	−1.63	0.1034
Time_Segment3 X Incongruent_blocks	−0.23	7.56	−14.95–14.50	−0.03	0.9759
Time_Segment4 X Incongruent_blocks	−0.09	7.99	−15.64–15.47	−0.01	0.9915
			**Variance**	**S.D**.	**Correlation**
**RANDOM EFFECTS**					
Participant			63,693.25	252.38	
Time_Segment1			4,642.83	68.14	−0.80
Time_Segment2			350.21	18.71	−0.48
Time_Segment3			15.21	3.90	0.48
Time_Segment4			28.76	5.36	−0.60
				**Marginal**	**Conditional**
**MODEL FIT R^2^**					
				0.42	0.92

Model equation: IES ~ (Time_Segment1 + Time_Segment2 + Time_Segment3 + Time_Segment4) ^*^Difficulty_of_blocks, random = ~ Time_Segment1 + Time_Segment2 + Time_Segment3 + Time_Segment4 | Participants, corAR1(0, form = ~ 1 | Participants)

* p < 0.05;

** p < 0.01;

*** *p < 0.001*.

### Effect on the Proximal Transfer Outcomes

**Figure 4:**
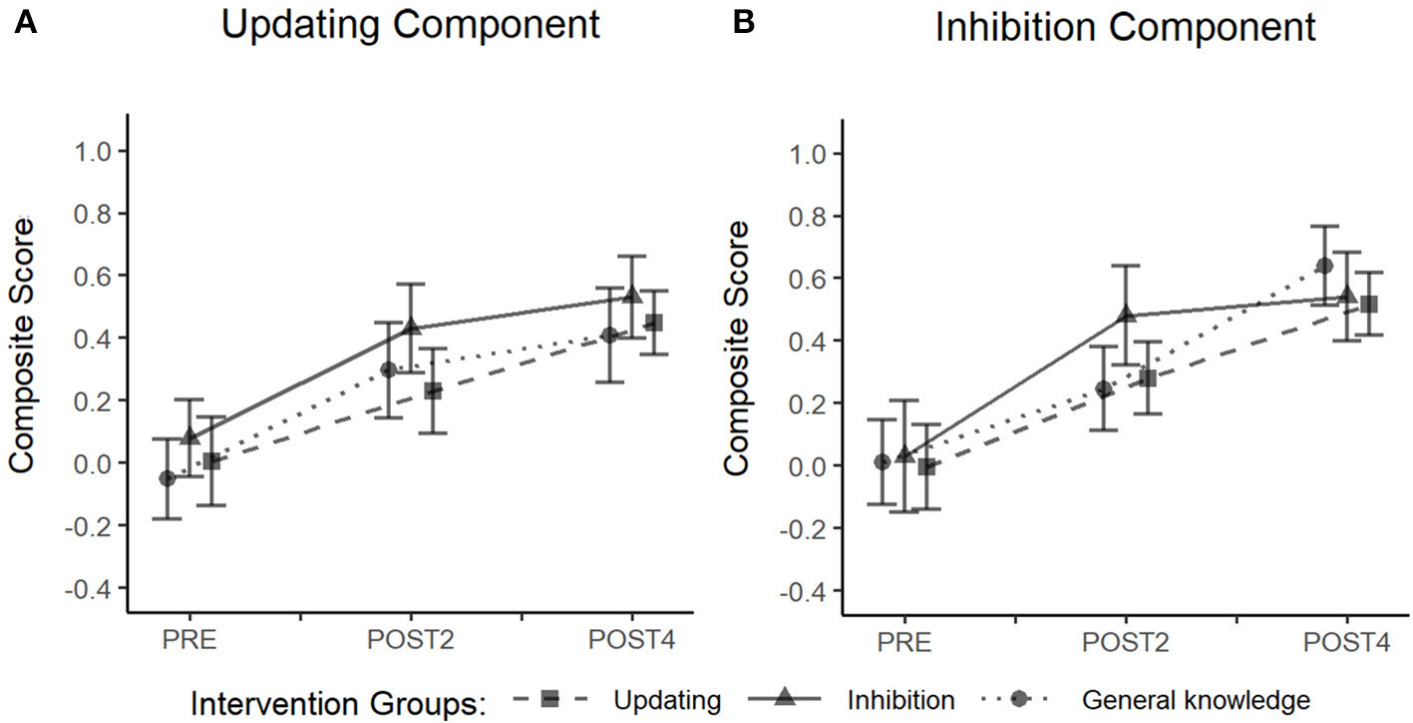
Performance growth on proximal transfer outcomes as a function of time and training group. Composite scores correspond to the averaged z scores of the antisaccade and Victoria Stroop tasks for the inhibition composite score **(A)**, and the averaged z scores of the keep track and running span tasks for the updating composite score **(B)**.

The building process of growth models showed significant unconditional growth of performance over time for the updating [χ^2^(1) = 26.32, *p* < 0.001] and inhibition [χ^2^(1) = 45.72, *p* < 0.001] composite scores (Figure 4). However, conditional growth models showed that the addition of the intervention group as a fixed effect and the interaction term, Time × Group, did not improve the fit of the model for neither the updating [χ^2^(2) = 0.87, *p* = 0.64, and χ^2^(4) = 0.91, *p* = 0.92, respectively], nor the inhibition composite score [χ^2^(2) = 0.21, *p* = 0.90, and χ^2^(4) = 0.80, *p* = 0.94, respectively]. A detailed presentation of the building process and results for the unconditional growth models are available in (Appendices 2, 3, respectively).

### Effect on the WM Transfer Outcomes

**Figure 5:**
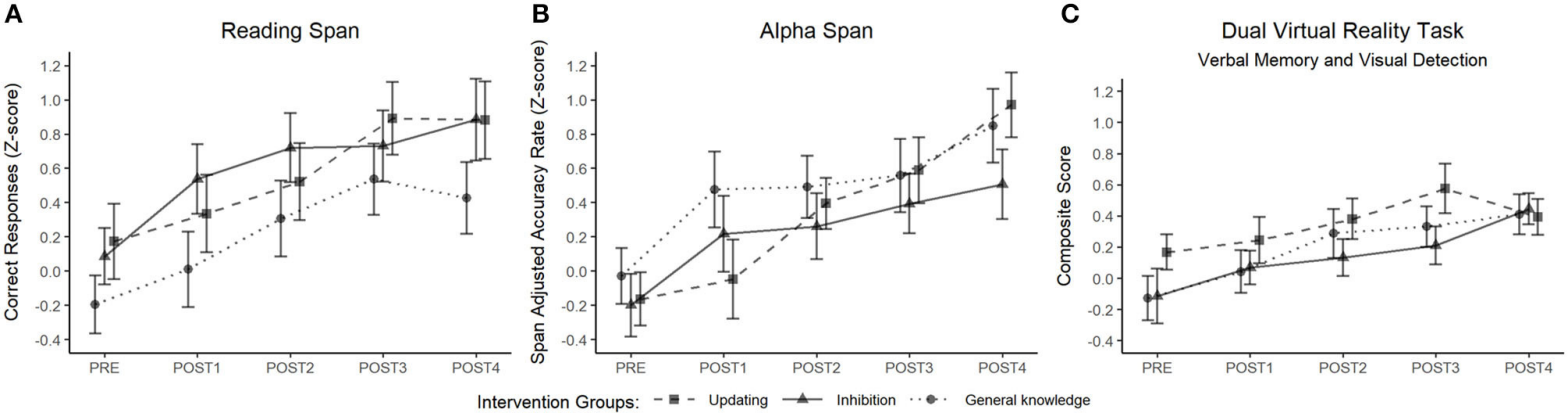
Performance growth on the complex WM outcomes as a function of time and intervention group. Correct responses in the reading span task **(A)** and accuracy rate in the alpha span task **(B)** are reported following a z score transformation. Composite scores in the dual virtual reality task **(C)** correspond to the mean between the z scores obtained on the verbal memory performance and the visual detection performance.

The building process of growth models showed a significant unconditional growth of performance over time on the reading span task [χ^2^(1) = 52.94, *p* < 0.001], alpha span task [χ^2^(1) = 44.73, *p* < 0.001], and dual virtual reality task [χ^2^(1) = 29.45, *p* < 0.001; Figure 5]. However, conditional growth models showed that the addition of the intervention group as a fixed effect and the interaction term, Time × Group, did not improve the fit of the model for any of the working memory tasks: reading span task [χ^2^(2) = 2.39, *p* = 0.30, and χ^2^(4) = 2.52, *p* = 0.64, respectively], alpha span task [χ^2^(2) = 1.44, *p* = 0.49, and χ^2^(4) = 4.93, *p* = 0.29, respectively] and dual virtual reality task [χ^2^(2) = 1.55, *p* = 0.46, and χ^2^(4) = 4.17, *p* = 0.38, respectively]. A detailed presentation of the building process and results for the unconditional growth models are available in (Appendices 2, 4, respectively).

### Age Group Comparison at POST4

The one-way ANOVAs computed for the proximal and WM transfer outcomes at POST4 are presented in Table 5. At POST4, the analyses showed that older adults had lower performance than untrained younger adults on the two composite scores (*post hoc* comparisons: all *p* < 0.05). They also showed lower performance on two of the complex WM transfer tasks, that are the alpha span task and the visual detection sub-task from the virtual car ride task (all *p* < 0.05). When examining performance on individual tasks, there was no effect of age on the keep track task (all *p* > 0.1), reading span task (all *p* > 0.1) and the visual detection sub-task from the virtual car ride task. In this last sub-task, the performance of the younger group was higher than that of the active control group (*p* < 0.05), but not the inhibition and updating training groups (all *p* > 0.1).

**Table 5:** Group comparison at POST4.

	**Updating intervention**	**Inhibition intervention**	**Active control intervention**		**Young adults (baseline)**	
**Characteristic**	***n* = 27**	***n* = 25**	***n* = 28**	***p*-values**	***n* = 30**	***p*-values**
**Inhibition composite measure**	0.2 ± 0.5	0.2 ± 0.7	0.3 ± 0.7	0.75	0.8 ± 0.4	0.00^***^
Victoria Stroop IF index (3rd plate/1st plate)	1.9 ± 0.3	1.9 ± 0.5	1.8 ± 0.4	0.70	1.6 ± 0.3	0.00^**^
Anti-saccade (range 0–90)	62.5 ± 21.4	62.9 ± 20.2	62.5 ± 21.4	0.93	78.1 ± 12.8	0.00^**^
**Updating composite measure**	0.2 ± 0.4	0.2 ± 0.6	0.1 ± 0.7	0.84	0.7 ± 0.8	0.00^**^
Keep track (range 0–24)	15.3 ± 2.1	15.4 ± 2.6	15.4 ± 3.4	0.99	16.2 ± 2.7	0.62
Running span (adjusted accuracy rate)	0.7 ± 0.1	0.7 ± 0.1	0.7 ± 0.1	0.66	0.8 ± 0.2	0.00^***^
**Complex working memory**						
Alpha span (adjusted accuracy rate)	0.8 ± 0.1	0.7 ± 0.1	0.8 ± 0.1	0.27	0.9 ± 0.1	0.00^***^
Reading span (range 0–56)	40.4 ± 9.7	40.4 ± 9.9	36.6 ± 9.2	0.25	42.0 ± 7.9	0.15
Dual virtual reality task composite measure	0.2 ± 0.6	0.1 ± 0.7	0.1 ± 0.6	0.92	0.8 ± 0.6	0.00^***^
Divided attention verbal memory (range 0–12)	4 ± 1.0	4.1 ± 1.0	4.4 ± 1.3	0.46	5.3 ± 1.4	0.00^***^
Divided attention visual detection (range 0–20)	18.1 ± 3.8	18.3 ± 1.9	16.4 ± 3.9	0.10	19.0 ± 1.6	0.02^*^

*P-value for analysis of variance group effect*.

* p < 0.05;

** p < 0.01;

*** *p < 0.001*.

## Discussion

The ACTOP study reports the first side-by-side comparison of the effect of updating and inhibition training on the cognition of older adults. The first objective was to examine how performance improves with repeated training and study the relationship between training dose and difficulty. The piecewise growth analysis models showed that performance increased the most (i.e., IES decreased) during the first time segment of both updating and inhibition training, which corresponds to the first three training sessions. This agrees with the rapid gains in performance observed in the early phases of training that involve repeated practice (21). Thereafter, the increase in performance was more gradual in inhibition training, and there was no increase observed in updating training in time segments 2, 3, and 4. However, these results do not take into account differences in levels of difficulty. Interestingly, we found that the dose effect was related to the difficulty level for updating training. Indeed, the slope of progression was more pronounced for the 3-back than 1-back condition during the first and the third time segment. As a result, the difference in performance between the 1-back and 3-back condition was reduced at the end of training. This suggests that updating training specifically improved updating capacities, as well as the ability to process other aspects of the tasks. The optimal dose, or the smallest dose needed to achieve maximal efficacy, seems to occur after ~9 training sessions. However, the slope of progression did not differ between the difficulty levels for inhibition training. The various conditions improved at the same rate, and the difference between the congruent and incongruent stimuli was not reduced at the end of the training. This may be due to a non-specific effect, insufficient dose or a floor effect.

The second objective was to examine whether the training effect generalized to proximal untrained tasks and complex WM tasks. Both inhibition and updating training yielded improvements in proximal updating and inhibition transfer measures, which is consistent with several cognitive interventions in older adults [e.g., (8, 10–14)]. The magnitude of the pre-post effect was comparable for the two training types. Similarly, both inhibition and updating training improved complex WM transfer tasks, including the virtual reality task, which suggests that WM performance can also be improved in tasks designed to mimic WM function in real-life situations. However, none of these pre-post effects differed from those observed in the active control training group. This could be due to the active control condition, which may involve attentional control. Indeed, even though we expected that the general knowledge training would have a limited effect on WM, the recruitment of high-level processes such as reasoning, encoding and retrieval of new information may have contributed to improve high-level WM capacities. An alternative explanation is that the improvements are mostly the result of repeated practice. This is supported by previous studies where transfer differences were observed between training groups. Because these studies incorporated transfer and training tasks sharing a very similar format and structure, equivalent strategies could be applied [e.g., (10–14)]. It is therefore possible that our findings differed from these prevous studies, as we incorporated tasks with differing structures to investigate the training-related transfer effect of updating and inhibition processes, rather than focus on learning strategies. It has been suggested that training basic processes, such as updating and inhibition, may not be the best approach to favor transfer. In a recent meta-analysis of the effect of computerized cognitive training on attentional control, Webb et al. (40) suggested that the path from basic to more complex abilities is not direct but may require multiple attention-demanding mental functions organized around top-down processing goals [also see Shipstead et al. (41)]. This would explain the challenge to observe transfer based on single-domain training [(9); see also Boujut and Belleville (42) for a review].

Finally, a critical objective was to determine whether training reduced the age effect by increasing the performance of older adults to the level of younger participants. Results were not consistent with a pervasive reduction of the age effect although there were some improvements. While there was an effect of age on all transfer tasks (proximal and complex WM) at baseline, the age difference was no longer found at POST4 for the keep track and reading span tasks, regardless of the training group.

### Limitations of the Study

One limitation is how attentional control training was not fully adaptive because changing the level of difficulty as the training sessions progressed would have prevented the measurement of the dose effect at each difficulty level. The piecewise segmentation in four time segments was arbitrary and may have influenced the pattern of performance growth. A simpler two-segment segmentation may have revealed a simpler pattern. Finally, it cannot be excluded that the large number of post-baseline evaluations may have reduced the effect of inhibition and updating training by contributing to practice effects on transfer tasks.

### Conclusion

To conclude, the overall results from this study suggest that attentional control training involving repeated practice is effective to improve updating and inhibition performances on training tasks. Nevertheless, we found that the dose effect was related to difficulty only for updating training. Here, the optimal dose to achieve efficacy is ~9:30-min training sessions, which is less than the maximal 12 sessions offered in this study. Despite an overall improvement of older adults on proximal and complex WM tasks, neither updating nor inhibition training provided additional improvements to the proximal and complex WM transfer tasks in comparison with the active control condition. This suggests that the efficacy of process-based training does not directly affect transfer tasks. It must be noted that these results were limited to behavioral data but it is possible that attentional control training in updating and inhibition leads to specific, measurable improvements in brain functioning.

## Data Availability Statement

The raw data supporting the conclusions of this article will be made available by the authors, without undue reservation.

## Ethics Statement

The studies involving human participants were reviewed and approved by Comité d'éthique de la recherche vieillissement-neuroimagerie Direction de l'enseignement universitaire et de la recherche (DEUR) CIUSSS du Center-Sud-de-l'Île-Montréal. The patients/participants provided their written informed consent to participate in this study.

## Author Contributions

SB is the leader of the trial. AB coordinated and oversaw all aspects of the project. Final decisions on the design were made by SB. AB and SB wrote the first version and the final revised version of the paper. All authors have participated in the conceptualization of the study, design, revised the paper, and accepted the final submitted version.

## Conflict of Interest

SB has been a consultant for research development on the prevention of Alzheimer disease for the Fondation IUGM (2016) and for Sojecci (2017 to current), and for the development of a cognitive stimulation program for the Centre de promotion de la Santé AvantÂge (2015). She has intellectual property rights on the “Programme de Stimulation pour une santé cognitive, Memoria, Batterie d'évaluation de la mémoire Côte-des-Neiges” and “MEMO, Méthode d'Entrainement pour une Mémoire Optimale.” She collaborates and receives funding from Mind Maze and Beam Me Up. The remaining authors declare that the research was conducted in the absence of any commercial or financial relationships that could be construed as a potential conflict of interest.
