# Supplementary material for: Effects of Computerized Updating and Inhibition Training in Older Adults: The ACTOP Three-Arm Randomized Double-Blind Controlled Trial
Source: Front Neurol. 2020 Dec 3;11:606873. doi: 10.3389/fneur.2020.606873 (PMC7744626; doi:10.3389/fneur.2020.606873)
Supplement: Supplementary file 1 [file Data_Sheet_1.docx]

Appendix 1. Model building process of the training performance growth.

|  | | | | | | |  | **Updating training performance** | | | | | |  | **Inhibition training performance** | | | | | |
| --- | --- | --- | --- | --- | --- | --- | --- | --- | --- | --- | --- | --- | --- | --- | --- | --- | --- | --- | --- | --- |
| Sampling Units | | | | | | |  | N total obs = 871  N Subjects = 29 | | | | | |  | N total obs = 600  N Subjects = 25 | | | | | |
| **Model specification** | **Model name** | **Nested / simpler Model** | **Fixed Effects added** |  | **Random Effects** | |  | **Model fit** | | | | **LRT Test against nested** | |  | **Model fit** | | | | **LRT Test against nested** | |
|  |  |  |  |  | **Subjects** | **Time** |  | **AIC** | **BIC** | **LL** | **df** | **df** | **X2** |  | **AIC** | **BIC** | **LL** | **df** | **df** | **X2** |
|  | | | | | | | | | | | | | | | | | | | | |
| Random Effect only | Null | - | - |  | intercepts | intercepts |  | 12362.5 | 12391.1 | -6175.3 | 6 |  |  |  | 7344.6 | 7370.9 | -3666.3 | 6 |  |  |
|  | | | | | | | | | | | | | | | | | | | | |
| Unconditional model | Linear | Null | Time |  | “ | slopes |  | 12329.3 | 12362.7 | -6157.6 | 7 | 1 | 35.267*** |  | 7302.5 | 7333.2 | -3644.2 | 7 | 1 | 44.108*** |
| Unconditional model | Quadratic | Linear | + I(Time^2) |  | “ | “ |  | 12259.7 | 12312.2 | -6118.9 | 11 | 4 | 77.527*** |  | 7174.3 | 7222.6 | -3576.1 | 11 | 4 | 136.202*** |
| Unconditional model | Piecewise | Quadratic | Segmented_Time |  | “ | “ |  | 12261.7 | 12366.6 | -6108.8 | 22 | 11 | 20.034* |  | 7092.1 | 7188.8 | -3524.1 | 22 | 11 | 104.144*** |
|  | | | | | | | | | | | | | | | | | | | | |
| Conditional model | Difficulty effect | Piecewise | + Difficulty |  | “ | “ |  | 11867.8 | 11982.2 | -5909.9 | 24 | 2 | 397.925*** |  | 6937.2 | 7038.4 | -3445.6 | 23 | 1 | 156.868*** |
| Conditional model | Interaction | Difficulty effect | + Segmented_Time x Difficulty |  | “ | “ |  | 11805.7 | 11958.3 | -5870.8 | 32 | 8 | 78.114*** |  | 6933.5 | 7052.2 | -3439.7 | 27 | 4 | 11.766* |

* *p*<.05; ** *p*<.01; *** *p*<.
