# Supplementary material for: Effects of Computerized Updating and Inhibition Training in Older Adults: The ACTOP Three-Arm Randomized Double-Blind Controlled Trial
Source: Front Neurol. 2020 Dec 3;11:606873. doi: 10.3389/fneur.2020.606873 (PMC7744626; doi:10.3389/fneur.2020.606873)
Supplement: Supplementary file 2 [file Data_Sheet_2.docx]

Appendix 2.

Model building process for the proximal transfer outcomes

|  | | | | | | |  | **Updating composite measure** | | | | | |  | **Inhibition composite measure** | | | | | |
| --- | --- | --- | --- | --- | --- | --- | --- | --- | --- | --- | --- | --- | --- | --- | --- | --- | --- | --- | --- | --- |
| Sampling Units | | | | | | |  | N total obs = 246  N Subjects = 83 | | | | | |  | N total obs = 241  N Subjects = 82 | | | | | |
| **Model specification** | **Model name** | **Nested / simpler Model** | **Fixed Effects added** |  | **Random Effects** | |  | **Model fit** | | | | **LRT Test against nested** | |  | **Model fit** | | | | **LRT Test against nested** | |
|  |  |  |  |  | **Subjects** | **Time** |  | **AIC** | **BIC** | **LL** | **df** | **df** | **X2** |  | **AIC** | **BIC** | **LL** | **df** | **df** | **X2** |
|  | | | | | | | | | | | | | | | | | | | | |
| Random Effect only | Null | - | - |  | intercepts | intercepts |  | 523.7 | 541.2 | -256.8 | 5 |  |  |  | 437.1 | 454.6 | -213.6 | 5 |  |  |
|  | | | | | | | | | | | | | | | | | | | | |
| Unconditional model | Time effect | Null | Time |  | “ | slopes |  | 499.4 | 520.4 | -243.7 | 6 | 1 | 26.317*** |  | 393.4 | 414.3 | -190.7 | 6 | 1 | 45.721*** |
|  | | | | | | | | | | | | | | | | | | | | |
| Conditional model | Group effect | Time effect | + Group |  | “ | “ |  | 502.5 | 530.5 | -243.3 | 8 | 2 | 0.887 *NS* |  | 397.2 | 425.1 | -190.6 | 8 | 2 | 0.206 *NS* |
| Conditional model | Interaction | Time effect | + Time x Group |  | “ | “ |  | 506.5 | 541.5 | -243.2 | 10 | 4 | 0.909 *NS* |  | 400.6 | 435.5 | -190.3 | 10 | 4 | 0.804 *NS* |

* *p*<.05; ** *p*<.01; *** *p*<.001; *NS =* not significant.

|  | | | | | | |  | **Reading span** | | | | | |  | **Alpha span** | | | | | |  | **Dual virtual reality task** | | | | | |
| --- | --- | --- | --- | --- | --- | --- | --- | --- | --- | --- | --- | --- | --- | --- | --- | --- | --- | --- | --- | --- | --- | --- | --- | --- | --- | --- | --- |
| Sampling Units | | | | | | |  | N total obs = 410  N Subjects = 83 | | | | | |  | N total obs = 410  N Subjects = 83 | | | | | |  | N total obs = 400  N Subjects = 80 | | | | | |
|  |  |  |  |  |  |  |  |  |  |  |  |  |  |  |  |  |  |  |  |  |  |  |  |  |  |  |  |
| **Model specification** | **Model name** | **Nested / simpler Model** | **Fixed Effects added** |  | **Random Effects** | |  | **Model fit** | | | | **LRT Test against nested** | |  | **Model fit** | | | | **LRT Test against nested** | |  | **Model fit** | | | | **LRT Test against nested** | |
|  |  |  |  |  | **Subjects** | **Time** |  | **AIC** | **BIC** | **LL** | **df** | **df** | **X2** |  | **AIC** | **BIC** | **LL** | **df** | **df** | **X2** |  | **AIC** | **BIC** | **LL** | **df** | **df** | **X2** |
|  | | | | | | | | | | | | | | | | | | | | | | | | | | | |
| Random Effect only | Null | - | - |  | intercepts | intercepts |  | 964.9 | 985.0 | -477.5 | 5 |  |  |  | 1119.9 | 1139.9 | -554.9 | 5 |  |  |  | 738.5 | 758.5 | -364.3 | 5 |  |  |
|  | | | | | | | | | | | | | | | | | | | | | | | | | | | |
| Unconditional model | Time effect | Null | Time |  | “ | slopes |  | 914.0 | 938.1 | -450.0 | 6 | 1 | 52.942*** |  | 1077.1 | 1101.2 | -532.6 | 6 | 1 | 44.734*** |  | 711.1 | 735.0 | -349.5 | 6 | 1 | 29.454*** |
|  | | | | | | | | | | | | | | | | | | | | | | | | | | | |
| Conditional model | Group effect | Time effect | + Group |  | “ | “ |  | 915.6 | 947.7 | -449.8 | 8 | 2 | 2.387 *NS* |  | 1079.7 | 1111.8 | -531.8 | 8 | 2 | 1.440 *NS* |  | 713.5 | 745.5 | -348.8 | 8 | 2 | 1.548 *NS* |
| Conditional model | Interaction | Time effect | + Time x Group |  | “ | “ |  | 919.5 | 959.6 | -449.7 | 10 | 4 | 2.516 *NS* |  | 1080.2 | 1120.3 | -530.1 | 10 | 4 | 4.933 *NS* |  | 714.9 | 754.8 | -347.5 | 10 | 4 | 2.620 *NS* |

Model building process for the WM transfer outcomes

* *p*<.05; ** *p*<.01; *** *p*<.001; *NS =* not significant.
