# Supplementary material for: Effects of Computerized Updating and Inhibition Training in Older Adults: The ACTOP Three-Arm Randomized Double-Blind Controlled Trial
Source: Front Neurol. 2020 Dec 3;11:606873. doi: 10.3389/fneur.2020.606873 (PMC7744626; doi:10.3389/fneur.2020.606873)
Supplement: Supplementary file 3 [file Data_Sheet_3.docx]

Appendix 3.

Linear mixed-effects model on updating composite measure (proximal transfer)

| Fixed Effects | | | | | | |
| --- | --- | --- | --- | --- | --- | --- |
|  | Est/Beta | SE | 95% CI | t | p | |
| Intercept | 0.03 | 0.07 | -0.11 - 0.18 | 0.45 | 0.65 *NS* | |
| Time | 0.23 | 0.04 | 0.14 - 0.31 | 5.32 | 0.0000*** | |
|  | | | | | | |
| Random Effects | | | | | | |
|  | | | Variance | S.D. | | Correlation |
| Participant | | | 0.20 | 0.45 | |  |
| Time | | | 0.00 | 0.01 | | -0.02 |
|  | | | | | | |
| Model fit | | | | | | |
| R^2^ | | | Marginal | Conditional | | |
|  | | | 0.07 | 0.44 | | |
| Model equation: Composite_score ~ Time * Difficulty, random = ~ Time \| Participants) | | | | | | |

* *p*<.05; ** *p*<.01; *** *p*<.001.

Linear mixed-effects model on inhibition composite measure (proximal transfer)

| Fixed Effects | | | | | | |
| --- | --- | --- | --- | --- | --- | --- |
|  | Est/Beta | SE | 95% CI | t | p | |
| Intercept | 0.00 | 0.09 | -0.16 - 0.17 | 0.05 | 0.96 *NS* | |
| Time | 0.29 | 0.04 | 0.22 - 0.36 | 7.89 | 0.0000*** | |
|  | | | | | | |
| Random Effects | | | | | | |
|  | | | Variance | S.D. | | Correlation |
| Participant | | | 0.51 | 0.71 | |  |
| Time | | | 0.06 | 0.24 | | -0.60 |
|  | | | | | | |
| Model fit | | | | | | |
| R^2^ | | | Marginal | Conditional | | |
|  | | | 0.10 | 0.83 | | |
| Model equation: Composite_score ~ Time * Difficulty, random = ~ Time \| Participants) | | | | | | |

* *p*<.05; ** *p*<.01; *** *p*<.001.
