# Supplementary material for: Effects of Computerized Updating and Inhibition Training in Older Adults: The ACTOP Three-Arm Randomized Double-Blind Controlled Trial
Source: Front Neurol. 2020 Dec 3;11:606873. doi: 10.3389/fneur.2020.606873 (PMC7744626; doi:10.3389/fneur.2020.606873)
Supplement: Supplementary file 4 [file Data_Sheet_4.docx]

Linear mixed-effects model on reading span task

| Fixed Effects | | | | | | |
| --- | --- | --- | --- | --- | --- | --- |
|  | Est/Beta | SE | 95% CI | t | p | |
| Intercept | 0.08 | 0.11 | -0.13 - 0.30 | 0.77 | 0.44 *NS* | |
| Time | 0.18 | 0.02 | 0.14 - 0.22 | 8.60 | 0.0000*** | |
|  | | | | | | |
| Random Effects | | | | | | |
|  | | | Variance | S.D. | | Correlation |
| Participant | | | 0.81 | 0.90 | |  |
| Time | | | 0.00 | 0.07 | | 0.42 |
|  | | | | | | |
| Model fit | | | | | | |
| R^2^ | | | Marginal | Conditional | | |
|  | | | 0.05 | 0.77 | | |
| Model equation: Z-score ~ Time * Difficulty, random = ~ Time \| Participants) | | | | | | |

* *p*<.05; ** *p*<.01; *** *p*<.001.

Linear mixed-effects model on alpha span task

| Fixed Effects | | | | | | |
| --- | --- | --- | --- | --- | --- | --- |
|  | Est/Beta | SE | 95% CI | t | p | |
| Intercept | -0.07 | 0.09 | -0.25 - 0.11 | -0.71 | 0.47 *NS* | |
| Time | 0.21 | 0.03 | 0.15 - 0.26 | 7.57 | 0.0000*** | |
|  | | | | | | |
| Random Effects | | | | | | |
|  | | | Variance | S.D. | | Correlation |
| Participant | | | 0.35 | 0.60 | |  |
| Time | | | 0.00 | 0.06 | | 0.56 |
|  | | | | | | |
| Model fit | | | | | | |
| R^2^ | | | Marginal | Conditional | | |
|  | | | 0.08 | 0.60 | | |
| Model equation: Z-score ~ Time * Difficulty, random = ~ Time \| Participants) | | | | | | |

* *p*<.05; ** *p*<.01; *** *p*<.001.

Linear mixed-effects model on dual virtual reality task

| Fixed Effects | | | | | | |
| --- | --- | --- | --- | --- | --- | --- |
|  | Est/Beta | SE | 95% CI | t | p | |
| Intercept | 0.01 | 0.07 | -0.13 - 0.16 | 0.19 | 0.85 *NS* | |
| Time | 0.11 | 0.02 | 0.07 - 0.15 | 5.94 | 0.0000*** | |
|  | | | | | | |
| Random Effects | | | | | | |
|  | | | Variance | S.D. | | Correlation |
| Participant | | | 0.31 | 0.56 | |  |
| Time | | | 0.00 | 0.07 | | -0.60 |
|  | | | | | | |
| Model fit | | | | | | |
| R^2^ | | | Marginal | Conditional | | |
|  | | | 0.05 | 0.55 | | |
| Model equation: Composite_score ~ Time * Difficulty, random = ~ Time \| Participants) | | | | | | |

* *p*<.05; ** *p*<.01; *** *p*<.001.
